# Linking Parenting and Social Competence in School-Aged Boys and Girls: Differential Socialization, Diathesis-Stress, or Differential Susceptibility?

**DOI:** 10.3389/fpsyg.2018.02789

**Published:** 2019-01-15

**Authors:** Andrea M. Spruijt, Marielle C. Dekker, Tim B. Ziermans, Hanna Swaab

**Affiliations:** ^1^Department of Clinical Child and Adolescent Studies, Faculty of Social and Behavioral Sciences, Leiden University, Leiden, Netherlands; ^2^Leiden Institute for Brain and Cognition, Leiden University, Leiden, Netherlands; ^3^Department of Psychology, University of Amsterdam, Amsterdam, Netherlands

**Keywords:** social cognition, social skills, gender differences, parent–child interaction, emotion recognition

## Abstract

Girls generally demonstrate superior skill levels in social competence compared to boys. The exact relations of parenting with these gender differences are currently unclear. Gender differences may occur due to exposure to different parenting strategies (differential socialization model) or due to a different impact of similar parenting strategies for boys and girls (differential susceptibility and diathesis-stress model).

**Objective:** In this study we assessed both hypotheses using a multi-method multi-informant approach. We investigated (1) to what extent different parenting strategies mediate the relation between gender and social competence and (2) whether gender and age moderate the relation between parenting strategies and social competence.

**Design:** Parenting strategies were observed during home visits and social competence was assessed using parent and teacher questionnaires and performance-based neurocognitive tasks (*N* = 98, aged 4 to 8).

**Results:** (1) Parenting strategies did not mediate the relation between gender and social competence. (2) Gender moderated the association between parental questioning style and children’s level of social competence: parents asking fewer questions was associated with poorer social cognitive skills in boys only. Parental supportive presence and intrusiveness were related to aspects of social competence irrespective of gender. Age moderated the relation between parenting and aspects of social competence, though in various (unexpected) directions.

**Conclusion:** Our findings do not support the differential socialization hypothesis and provide partial evidence for a diathesis-stress model as an explanation for parental influence on gender differences in social competence.

## Introduction

When children start school their world begins to open up as they increasingly interact with children and adults outside their family. The new school environment requires adaptive social skills in order to build friendships, learn to cooperate, and optimally benefit from learning opportunities. The cognitive, emotional, and social skills necessary for effective social interactions can be described as social competence (Kostelnik et al., 2014). Social competence is particularly important at school entry and in the first few years of school, when social interactions are critical for academic success (Raver, 2002). Social competence has repeatedly been linked to school performance (e.g., Shala, 2013) and is considered to be as important for school success as academic skills are (Raver and Zigler, 1997; NICHD, 2004). Parents play a crucial role herein as parent–child interaction is considered the foundation on which social development is built (Laible and Thompson, 2007).

Social cognition can be described as the neurocognitive mechanisms underlying social competence, including the ability to interpret, predict, and empathize with others’ mental states and behaviors (Baron-Cohen et al., 1999). According to the social information processing approach (Crick and Dodge, 1994), several successive social cognitive steps are taken to interpret and adequately respond to each new social situation. The child first focuses on specific social cues, such as facial expressions, and interprets these cues within the social context. The child then considers and evaluates possible responses to this situation from his own personal database, based on past experiences. Finally, this database is used as a guide to choose the perceived most adequate response. This process is iterative and is highly influenced by the social environment, as the personal database is constantly updated with the most recent social encounters. Lemerise and Arsenio (2000) argued that social information processing cannot be seen separately from emotion processes and therefore proposed a revised model into which emotion processes are integrated and can both influence and be influenced by each step of social information processing. For instance, mood and social situation influence how social cues are interpreted and responses are evaluated. Children with better social information processing skills have been found to be more socially competent, both in preschool (Ziv, 2013) and in primary school (Mayeux and Cillessen, 2003).

The development of social cognitive and behavioral skills originates within the relationship with the child’s parents or significant caregivers (Vygotsky, 1978; Attili et al., 2010). Parents provide their child with early social learning opportunities and are responsible for communicating social rules to their children, supporting the development of a database of adequate social behavior (Bennett et al., 2005). When children start school, these skills are necessary to build adequate relationships with peers and form friendships. In turn, having prosocial friends can promote social competence (Wentzel et al., 2004). Aspects of parental sensitivity, such as parental support and intrusiveness, have repeatedly been found to predict the development of social competence (e.g., Lengua et al., 2007; Spinrad et al., 2007; Barnett et al., 2012). Parental support refers to warm and affective caregiving, while intrusiveness refers to negative and controlling parenting or lack of autonomy support (Dotterer et al., 2012). In addition, the manner in which parents verbally interact with their children, e.g., through scaffolding and asking open-ended questions, helps children to practice their communication skills, which in turn promote social development (Vygotsky, 1978; Gallagher, 1993; Lee et al., 2012). Even though there is compelling evidence relating parental scaffolding to children’s cognitive abilities and school achievement, studies focusing on the association between scaffolding and social development are scarce (for a review, see Mermelshtine, 2017).

The development of social understanding can be described by successive social cognitive stages and largely takes place between 4 years of age and adolescence (Selman, 1980, 2003). Even though there is a gradual increase in social understanding with increasing age (e.g., Marcone et al., 2015), the presence of great individual variability among same-aged children persists. In particular, during middle childhood quite robust gender differences in social understanding favoring girls have been found (e.g., Abdi, 2010; for a meta-analysis, see Fabes and Eisenberg, 1998). Girls tend to develop their social information processing skills more rapidly, which allows them to interpret and learn from social interactions at an earlier age than boys do (Bennett et al., 2005). For instance, especially during infancy and the preschool period, girls have been found to outperform boys in facial emotion processing (for a meta-analysis, see McClure, 2000) and emotion knowledge (Denham et al., 2015). Gender differences in social competence may be explained by parents using different parenting strategies toward sons and daughters, assuming a differential socialization model (Lytton and Romney, 1991). Alternatively, differences in social functioning can be considered a result of a differential impact of parenting strategies on social development for boys and girls (Rutter et al., 2003).

The differential socialization model assumes that parenting strategies may mediate the relation between gender and level of social competence. Girls may elicit different responses from their social environment than boys. This might for example be due to different social expectations or as a result of their more mature skills. For instance, parents may initiate more or other types of verbal interaction with their daughters because they are more responsive (Leaper, 2002). Leaper et al. (1998) showed in their meta-analysis that mothers were more talkative with their daughters than with their sons. In addition, research shows that parents talk more about emotions with their 4-year-old daughters than with their sons (Fivush et al., 2000; Aznar and Tenenbaum, 2015). In turn, more emotion talk predicts emotion understanding, an important aspect of social cognition, 6 months later in 4- to 6-year-olds (Aznar and Tenenbaum, 2013).

However, gender-differentiated socialization remains debated, and might only be true for some aspects of parenting in relation to social competence (for a review, see Leaper, 2002). In a recent meta-analysis focusing on parental sensitivity (Endendijk et al., 2016), it was concluded that differences in parenting of boys and girls are minimal, in line with an earlier meta-analysis by Lytton and Romney (1991). Leaper et al. (1998), who did find differences in parenting sons and daughters, argued that the discrepancy between Lytton and Romney’s findings and their own may be due to the broadly defined parenting behaviors used by the former (e.g., amount of interaction, undifferentiated), possibly obscuring differential socialization of girls and boys. An additional explanation may be the focus on different aspects of parenting in these meta-analyses. Where Leaper et al. (1998) specifically focused on verbal interaction such as amount of talking and supportive and directive speech, Endendijk et al. (2016) examined parental support and intrusiveness. These findings suggest gender-differentiated parenting may only be true for some, but not all parenting strategies that have been associated with social competence and emphasizes the need to study different parenting strategies simultaneously.

Alternatively, a differential impact of environmental influences has been suggested to explain gender differences in child behavior (Rutter et al., 2003). This suggests boys and girls are exposed to similar parenting strategies, but that these strategies have different effects on their social and behavioral development. In other words, gender acts as a moderator in the relation between parenting strategies and social competence. The diathesis-stress model states that some individuals are more vulnerable to poor environmental experiences, such as low quality parenting, and will show worse developmental outcomes than individuals who are less vulnerable (Heim and Nemeroff, 1999). Gender may be a factor that distinguishes children who are more vulnerable to some environment-outcome relations from those who are not (e.g., Belsky, 2013). Research on the development of child behavioral problems supports this model, suggesting boys are more vulnerable to the negative effects of environmental adversity than girls (e.g., Calkins, 2002; Crick and Zahn-Waxler, 2003; Barnett and Scaramella, 2013). For example, Calkins (2002) reported that more parental intrusiveness was associated to emotional distress only in boys, and less maternal sensitivity has been found to predict more externalizing behavior in 9-year-old boys but not in girls (Miner and Clarke-Stewart, 2008).

However, positive parenting has also been related to fewer externalizing behaviors in boys but not in girls (for a review, see Rothbaum and Weisz, 1994); and positive parent–child interactions have been linked to fewer emotional problems only in boys (Browne et al., 2010). This is in line with a differential susceptibility model, which assumes that some individuals are not only more vulnerable to adverse environments (diathesis-stress), but that sensitivity to both negative and positive environments is enhanced (for reviews, see Belsky and Pluess, 2009; Ellis et al., 2011). In other words, some children are more susceptible to both positive (e.g., supportive presence and asking more open-ended questions) and negative (e.g., intrusiveness and asking fewer questions) aspects of parenting, which in turn leads to either the best or the worst developmental outcomes. In contrast, children who are relatively less affected by environmental influences will thrive less under optimal parenting conditions, but will also be less affected under adverse parental influences. Consistent with this differential susceptibility perspective, the association between sensitive parenting and social competence may also be stronger for boys than for girls (for a review, see Bornstein, 2005). For instance, maternal emotion talk has been found to predict 3-year-old boys’ but not girls’ emotion understanding, while there were no gender differences in amount of emotion talk, nor in their emotion understanding (Martin and Green, 2005). This suggests that in the case of emotion understanding, one of the elements of social competence, gender acts as a resiliency factor to the influence of parent–child interaction.

Age may also play a moderating role in the association between parenting and aspects of social competence, as parents adapt their expectations and parenting to their child’s age, and as individual differences in environmental susceptibility may vary with age (Ellis et al., 2011; Barnett and Scaramella, 2013). Furthermore, the nature of the relation between parenting strategies and child behavior may shift with age (Bradley et al., 2015; Spruijt et al., 2018). For instance, parental directiveness (i.e., providing verbal structure) has been shown to have a positive effect on cognitive and social development, but this effect reverses after age four, in line with the child’s diminished need for structure (Landry et al., 2000). When children grow-up and enter school, the school environment becomes increasingly important in providing a new setting to practice social skills with peers, relative to the impact of parenting. While children’s emotion understanding and perspective taking abilities develop rapidly during the transition to school (Wellman, 2007; Harris et al., 2016) and the influence of peers on social development increases (Rubin et al., 2013), the relative influence of parents on the development of social functions will likely decrease (Flynn, 2007), suggesting a moderating effect of age. These findings suggest that boys’ and girls’ differential susceptibility to various parenting strategies may also change with age.

In the current study, we aimed to investigate whether different aspects of parenting strategies (e.g., parental support, intrusiveness, and the amount and type of questions parents ask their children) are associated with various aspects of children’s social competence (social cognition, social behavioral competence at home and at school) during the early school years. We examined to what extent (1) these parental strategies mediate the relation between gender and social competence, substantiating the differential socialization model and (2) whether gender and age moderate these relations, substantiating the differential susceptibility or the diathesis-stress model. As both latter models posit statistical moderation, we will follow the recommendations proposed by Roisman et al. (2012) for distinguishing the differential susceptibility model (for better and for worse) from the diathesis-stress model (only for worse). Since social competence is linked to verbal ability (Gallagher, 1993; Milligan et al., 2007) and gender differences in verbal ability have been found (Toivainen et al., 2017), the current study evaluated whether associations were independent of children’s verbal ability.

We expect a mediated effect of gender on social competence through *(i)* parental questioning style but not *(ii)* parental support and intrusiveness. This indirect effect would support the differential socialization model for parental questioning style. It is expected that parents ask more questions to their daughters than to their sons (Leaper et al., 1998), which results in girls to outperform boys in social competence. In contrast, aspects of parental sensitivity are not expected to differ much for boys and girls in general (Lytton and Romney, 1991; Endendijk et al., 2016). Furthermore, we expect that the relative influence of parenting strategies on social competence will be moderated by gender. We hypothesize that boys are more susceptible to both *(iii)* positive (i.e., supportive presence and asking more (open-ended) questions) and *(iv)* negative [i.e., intrusiveness and asking less (open-ended) questions] aspects of parenting with regard to their social competence (Bornstein, 2005), which would support the differential susceptibility model. Furthermore, we expect that the relative influence of parenting strategies on social competence will *(v)* decrease with age (Landry et al., 2000; Flynn, 2007).”

## Materials and Methods

### Participants

The current study is embedded within the ‘Leiden Curious Minds Research Program’: a longitudinal program investigating the development of executive and social functioning in primary school children in the Netherlands and the effects of a parent and a teacher intervention program [approved by the Ethical Board of the Department of Education and Child Studies at Leiden University (ECPW-2010016)].

Parents of 4- to 8-year-old children from the lowest four grades of two Dutch primary schools (pre-school to second grade in United States school system), from towns that are part of the urban agglomeration of Rotterdam and the conurbation of The Hague, agreed to participate in this study by signing an informed consent letter. The current study uses child, paper-and-pencil tests to assess level of social cognitive skills and verbal ability, parent- and teacher reported social behavioral skills reports, and observational data on parents’ interactive behavior with their child collected during a home visit. Parents of 99 out of 138 children agreed to a home visit (response = 71.7%; 10.1% fathers). Participants whose parents agreed to a home visit did not significantly differ from those who did not agree to a home visit by age, gender, school, grade, or referral to mental health care in the past year, nor did their families differ in single parenthood status or parental education. One child refused to complete the neurocognitive assessments and was excluded from analyses (Final *N* neurocognitive assessments = 98). Information on social behavioral skills was missing for seven children due to non-response on the parental questionnaire (Final *N* parent questionnaire = 91) and for nine children due to non-response on the teacher questionnaire (Final *N* teacher questionnaire = 89). Children ranged in age from 4 to 8 years (*M* = 6.2 years, *SD* = 1.2) and 56.1% were male. No parents or children were excluded because of problems with oral or written proficiency in Dutch. For detailed sample characteristics, see Table 1.

**Table 1:** Participant characteristics (*N* = 98) and descriptive statistics of variables of interest.

	Total	Boys	Girls
*N*	98	55	43
Age in months [*M (SD*)]	74.30 (14.56) [49–101]	74.76 (14.91) [49–101]	73.42 (14.01) [49–101]
**Parental education (%)^a^**			
High	40.43	42.59	37.50
Medium	52.13	46.29	60.00
Low	7.45	11.11	2.50
Single parenthood (%)	6.38	7.41	5.00
**Parental sensitivity^b^**			
Supportive presence	3.95 (1.46) [1.00–6.75]	4.03 (1.39) [1.50–6.75]	3.84 (1.55) [1.00–6.75]
Intrusiveness	3.76 (1.42) [1.00–7.00]	3.75 (1.45) [1.00–6.50]	3.78 (1.38) [1.50–7.00]
**Number of questions per minute^b^**			
Total questions	4.19 (1.63) [0.17–9.27]	4.25 (1.61) [1.47–9.27]	4.12 (1.67) [0.17–7.36]
Closed-ended questions	2.16 (0.94) [0–4.19]	2.16 (0.93) [0.64–4.90]	2.16 (0.95) [0–4.66]
Open-ended questions	1.86 (0.95) [0.17–5.18]	1.90 (0.92) [0.43–5.18]	1.80 (0.99) [0.17–4.24]
**Child social competence**			
Social behavior at school	45.33 (10.46) [20.00–60.00]	42.00 (10.43) [20.00–59.00]	49.59 (8.93) [28.00–60.00]
Social behavior at home	55.10 (9.83) [29.00–75.00]	53.06 (9.54) [35.00–75.00]	57.95 (9.64) [29.00–71.00]
Social cognition	28.77 (14.72) [0–63.00]	27.62 (14.80) [0–61.00]	30.23 (14.66) [2.00–63.00]

*All data are presented as Mean (Standard deviation), [range] unless otherwise noted. ^a^Background information was missing for n = 4 children due to non-response on parental questionnaires. ^b^Original values before standardization.*

### Procedure

Paper-and-pencil and computer-based performance tasks were administered in a separate room at the child’s school, during two individual test sessions of approximately 60 min each. After each session the children could choose a small present as a token of appreciation. One session included fixed-order paper-and-pencil tasks and the other session mainly consisted of fixed-order computer tasks. Tests were administered by two trained Master’s students or by one of the main investigators (AMS, MCD). All home visits were conducted by Master’s student pairs. Test data were collected in the period between November 2013 and February 2014 (school 1) and between May and June 2014 (school 2).

### Measures

#### Demographic Characteristics

Parents were asked to fill out a complementary background information questionnaire, using the online survey software Qualtrics^1^. The highest completed level of education by the parent who participated in the home visit was used as an measure of educational attainment, according to the Dutch Standard Classification of Education (SOI), which is based on UNESCO’s International Standard Classification of Education (ISCED) [“SOI, 2003 (Issue 2006/’07),”]: (1) primary education (SOI levels 1 to 3; at most vocational training); (2) secondary education (level 4 of SOI); and (3) higher education (levels 5 to 7 of SOI; bachelor’s degree or higher). Single parenthood status was defined by not having the child’s other parent or a new caregiver living in the same household. Mental health care referrals were assessed by asking parents whether their child had been referred, examined or treated for emotional and behavioral problems in the past year.

#### Verbal Ability

Verbal ability was measured with the Concepts and Following Directions task of the Clinical Evaluation of Language Fundamentals (CELF-4NL) (Semel et al., 2010). This task gives an indication of the child’s ability to interpret and act upon spoken directions of increasing length and complexity. Among several choices, participants were asked to point out the pictured objects that were mentioned, requiring them to remember the names, characteristics and order of mention. Administration took approximately 20 min. The task contains 49 items of increasing length and complexity. Upon reaching item 19, the task was aborted after seven consecutive incorrect answers. Administered items were afterward coded to yield either 0 points for an incorrect answer or 1 point for a correct answer. Summed raw scores were used in the analyses. The test–retest reliability (*r* = 0.76) of this subtask is considered sufficient (Semel et al., 2010).

#### Social Competence

##### Social behavioral competence

Parents and teachers were also asked to fill out the Social Skills Rating System (SSRS) to measure social skills at home or at school (Gresham and Elliott, 1990; Van der Oord et al., 2005). Parents filled out the SSRS questionnaire using the online survey software Qualtrics^1^, while teachers filled out a hardcopy version. The SSRS has satisfactory internal consistency, test–retest reliability and convergent and discriminant validity, and is used for children from 3 to 18 years old. The teacher and parent version of the SSRS are rated on a three-point Likert scale ranging from 0 (never) to 2 (often). The SSRS teacher version consists of three subscales with 10 items each. The subscale “cooperation” assesses behavior like helping others. The subscale “assertion” assesses initiating behaviors such as asking for information. The subscale “self-control” assesses behavior like responding in conflict situations and taking turns. A sample item of this scale is “responds appropriately when pushed or hit by other children.” The three subscales form a total social skills scale score, with a range of 0–60. The Cronbach’s alpha for the teacher version of the SSRS in this sample was 0.93. The SSRS parent version consists of 4 subscales of 10 items each. In addition to the subscales “cooperation,” “assertion,” and “self-control,” the parent version also contains the subscale “responsibility.” A sample item of this scale is “requests permission before leaving the house.” The four subscales form a total social skills scale score (range from 0 to 80). The Cronbach’s alpha for the parent version of the SSRS in this sample was 0.89. The total raw score on each questionnaire was used in the analyses. A higher score indicates better social skills.

##### Social cognitive competence

Social cognition was measured with two parallel versions (A or B) of the short form of the Social Cognitive Skill Test (SCST) (Van Manen, 2007). The SCST is a semi-structured interview, based on the structural developmental approach of social cognition as proposed by Selman and Byrne (1974). Participants completed either version A or B, corresponding their randomly assigned A or B condition during the home visit. Both versions consisted of three short stories with accompanying pictures depicting different social situations in which a child is confronted with a problem. Administration took approximately 20 min. Eight questions regarding emotion recognition and perspective taking, increasing in difficulty, were asked per story, which were afterward coded to yield either: (i) 3 points; when the answer was correct straightaway; (ii) 1 point; when the answer was not completely correct, but after a supplementary question became correct; (iii) 0 points; when the answer was incorrect from the start or still not completely correct after a supplementary question. A story was aborted after two consecutive incorrect answers. Summed raw scores were used in the analyses. The correlation between version A and B has been shown to be 0.84 with test–retest reliability ranging from 0.77 for version A to 0.78 for version B (Van Manen, 2007).

#### Parenting Strategies

Parent’s interactive behavior with their child was videotaped during a home visit, while each parent–child dyad was engaged in two joint activity tasks of approximately 5–10 min. These tasks consisted of a sorting task and a combining task based on tasks designed by Utrecht University (Corvers et al., 2012). Parent–child dyads were randomly assigned to either complete task version A (*N* = 50, 51%) or task version B of each joint activity task (*N* = 48, 49%). Version A of either task included sorting different types of toy animals and combining four different eyes and four different mouths to form smiley faces with various facial expressions, and version B consisted of sorting different types of toy food and combining four different flower petals with four different disks to form unique flowers. Parent–child dyads were free to sort and combine the items according to their own strategy, as long as all combinations in the combining task were different. Parents were instructed to assist their child as they would normally do. The videotapes were coded afterward for global level of parental supportive presence and intrusiveness, as well as the amount of and different form of questions (i.e., open- or closed-ended) asked by the parent.

##### Parental supportive presence and intrusiveness

Parental support and intrusiveness were coded using the revised Erickson seven-point scale for Supportive presence (SP) and Intrusiveness (Egeland et al., 1990, Unpublished). A parent scoring high on SP shows emotional support to the child and is reassuring when the child is having difficulty with the task. A parent scoring high on Intrusiveness lacks respect for the child’s autonomy and does not acknowledge the child’s intentions or desires. Three coders who were blind to other data concerning the child or the parent coded the joint activity tasks. For each parent–child dyad, the combining and sorting task were coded independently by different coders. All coders completed an extensive training, consisting of several practice and feedback sessions supervised by an expert coder. Reliability of the coders [intraclass correlation (ICC)] was assessed directly after completion of the training and at the end of the coding process to detect possible rater drift. ICCs between coders directly after training were 0.92 for the SP scale (*N* = 12) and 0.81 for the Intrusiveness scale (*N* = 12). At the end of the coding process, ICCs were 0.91 for the SP scale (*N* = 12) and 0.92 for the Intrusiveness scale (*N* = 12), suggesting no significant rater drift. Whenever interactions were difficult to score due to an ambiguous interaction (*N* = 14), consensus was sought after a discussion with all coders. Even though parent–child dyads were randomly assigned to either joint task battery A or B, each task battery may have elicited a somewhat different interaction between parent and child. Therefore, level of SP and Intrusiveness was computed by standardizing each task version score (A or B) within each task (sorting or combining), followed by averaging these Z-scores over both joint activity tasks.

##### Parental questioning style

The total number and form of questions parents asked their children during the joint activity tasks were coded from video recordings using transcribed verbatim reports. Each question was coded as either being (i) open-ended (e.g., “How do you want to start?”; (ii) multiple choice (e.g., “Does a kangaroo live in the zoo or in the ocean?”; or (iii) closed-ended (e.g., “Is a cow a farm animal?”). The form of each question was coded by three coders who were not involved in coding SP and Intrusiveness and who were blind to other data concerning the child or the parent. All coders completed an extensive training, consisting of several practice and feedback sessions supervised by the main researcher. Interrater reliability (Cohen’s kappa) was high, with 0.84 on average for the sorting task (*N*_questions_ = 122) and 0.87 on average for the combining task (*N*_questions_ = 115). Within each task the number of questions per minute was calculated. Even though parent–child dyads were randomly assigned to either joint task battery A or B, each task battery may have elicited a somewhat different interaction between parent and child. Therefore, we standardized the number of questions per minute within each task (sorting or combining) for each task version (A or B), followed by averaging these Z-scores over the joint activity tasks. Due to very low occurrence of multiple-choice questions (2.4%), this form was excluded from further analyses. The difference score between the standardized amounts of open- and closed-ended questions was calculated as a relative measure of question format preference during the tasks. A higher open- versus closed-ended ratio score indicates that the parent asked more open-ended than closed-ended questions relative to the other parents. Total number of questions and open- versus closed-ended ratio score per minute were used as measures for parental questioning style.

### Data Analyses

**FIGURE 1:**
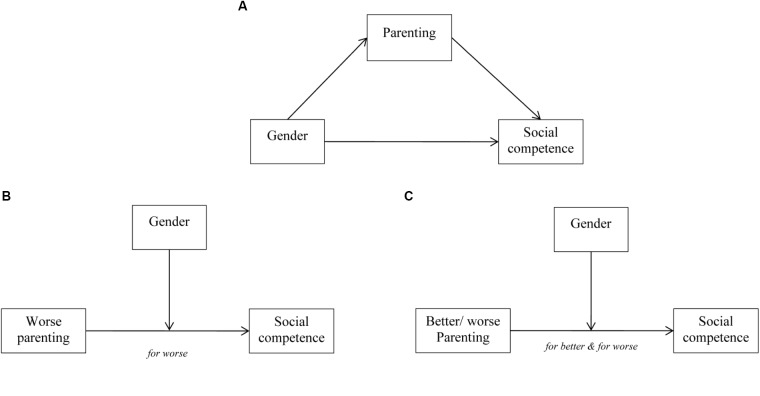
**(A)** Graphical representation Differential socialization model (mediation model). **(B)** Graphical representation Diathesis stress model (moderation model). Social competence is only related to gender at the low end (−2 SD) of the distribution of parenting. **(C)** Graphical representation Differential susceptibility model (moderation model). Social competence is related to gender at both high (+2 SD) and low ends (−2 SD) of the distribution of parenting.

Data were analyzed using IBM SPSS version 23. Demographic characteristics for both schools were compared with chi-square tests, independent *t*-tests and Fisher exact tests. Bootstrapping, a non-parametric resampling procedure recommended for small samples, was used to test the mediational models (Preacher and Hayes, 2004; Hayes, 2009). Bootstrapping with 5000 resamples was done to test for significant indirect effects, using an SPSS macro developed by Hayes (2012). Verbal ability and age were controlled for in all analyses. In this analysis, mediation is significant if the 95% bias corrected and accelerated confidence intervals for the indirect effect do not include 0 (Preacher and Hayes, 2004; Hayes, 2013). Separate hierarchical linear regression analyses were performed to assess whether each parenting strategy (independent variables) explained additional variance in each aspect of social competence (dependent variables) above or in interaction with gender and age, while controlling for verbal ability. Age and verbal ability were centered and all aspects of parenting were standardized to z-scores. In each regression analysis the following models were tested: (model i) the aspects of parenting strategy, verbal ability, gender, and age were included; (model ii) the quadratic term of parenting strategy was added to test for non-linearity (Roisman et al., 2012) and avoid misleading interactions (Ganzach, 1997); (model iii) the interaction term between parenting strategy and gender was added; (model iv) the interaction between parenting strategy and age was added; (model v) the interaction between gender and age and the three-way interaction between parenting strategy, gender and age were added. *F* for change in *R^2^* was used to assess whether a more extensive model significantly improved the amount of variance explained in comparison with a previous nested and more parsimonious model. Predicted *R^2^* was computed as a cross-validation measure. A negative predicted *R^2^* or a sizeable difference between predicted and regular [(adjusted) *R^2^*] can be an indication of an overfitted model (i.e., predicting random noise). Significant interaction models were also examined by calculating the posterior probability favoring the alternative hypothesis (i.e., evidence for an interaction effect) using the JZS Bayes Factor (BF_10_, calculated with Rouder’s web based application at http://pcl.missouri.edu/bayesfactor), which provides the odds ratio for the alternative/null hypotheses given the data (where 1 means that they are equally likely, larger values indicate more evidence for the interaction effect, and values below 1 indicate more evidence for the null hypothesis. Values between 1 and 3 are considered anecdotal evidence for the alternative hypothesis and values between −3 and 1 for the null, respectively) (Rouder and Morey, 2012). Significant interactions were consecutively probed with regression analyses that included a conditional moderator variable (e.g., low-age: 1 *SD* below *M*_age_; and high-age: 1 *SD* above *M*_age_ or: male; and female) (Holmbeck, 2002). Regression lines were plotted based on the resulting regression equations and significance of *t*-tests were reported for each simple slope. Regions of Significance (RoS) tests were conducted (Preacher et al., 2006) whenever a significant moderation effect for gender was found, in order to differentiate between a diathesis-stress and differential susceptibility model. This way it was analyzed if Y (social competence) and Z (gender) are related at both low and high ends (±2 *SD*) or only at the low end (−2 *SD*) of the distribution of X (parenting strategies), as recommended by Roisman et al. (2012). A graphic representation of all models is supplied in Figure 1. For all significant linear effects, standardized beta coefficients addressed effect size (0.2 = small effect; 0.5 = moderate effect; 0.8 = strong effect (Ferguson, 2009). Alpha for significant effects was set at *p* < 0.05.

## Results

Sample characteristics and descriptive statistics for the variables of interest are displayed in Table 1. The educational level and single parenthood status for parents of sons did not significantly differ from those for parents of daughters. Schools did not significantly differ on background characteristics of the participants: age (*p* = 0.63), gender (*p* = 0.13), single parenthood status (*p* = 0.16), parental education (*p* = 0.07), or prevalence of referral to mental health care in the past year (*p* = 0.93). Simple correlations between all independent and dependent variables and partial correlations controlled for verbal ability are presented in Table 2.

**Table 2:** Intercorrelations among all variables.

	1	2	3	4	5	6	7	8	9	10	11
(1) Age	–	−0.05	−0.11	0.20^∗^	−0.41^∗∗^	0.05	0.05	0.02	0.62^∗∗^	0.01	0.08
(2) Gender	−0.05	–	−0.05	< −0.01	<-0.01	−0.05	0.36^∗∗∗^	0.25^∗^	0.09	0.03	−0.05
(3) Supportive presence	< −0.01	−0.05	–	−0.80^∗∗^	0.27^∗∗^	0.15	0.11	−0.02	0.06	0.29^∗∗^	−0.04
(4) Intrusiveness	0.08	< −0.01	−0.85^∗∗^	–	−0.13	−0.18^†^	−0.24^∗^	−0.07	−0.04	−0.31^∗∗^	0.13
(5) Total questions	−0.06	−0.02	0.16	< −0.01	–	−0.04	−0.18^†^	0.02	−0.22^∗^	−0.06	−0.13
(6) Ratio open-closed questions	−0.01	−0.05	0.18	−0.27^∗^	0.01	–	−0.03	0.14	0.05	0.10	<0.01
(7) Social behavior at school	−0.17	0.34^∗∗^	0.19^†^	−0.25^∗^	−0.10	0.06	–	0.24^∗^	0.15	0.32^∗∗^	−0.10
(8) Social behavior at home	−0.14	0.23^∗^	−0.01	−0.08	0.08	0.16	0.21^†^	–	0.11	0.24^∗^	0.02
(9) Social cognition	0.34^∗∗^	0.13	0.16	−0.17	0.05	<0.01	< 0.01	0.01	–	0.08	0.01
(10) Parental education	−0.14	0.03	0.31^∗∗^	−0.33^∗∗^	0.01	0.10	0.26^∗^	0.23^∗^	−0.01	–	−0.07
(11) Single parenthood	0.22^∗^	−0.05	−0.06	0.14	−0.19^†^	0.01	−0.10	−0.01	0.08	−0.06	–

*Bivariate correlations among all variables above the diagonal; below the diagonal are partial correlations, controlling for verbal ability. ^†^p < 0.10; ^∗^p < 0.05; ^∗∗^p < 0.01; ^∗∗∗^p < 0.001.*

### Mediation Analyses: Differential Socialization

Bias-corrected bootstrapping analyses were conducted to test for an indirect effect of gender on social competence (social behavioral competence (i) at school and (ii) at home, and (iii) social cognition) through parenting strategies (parental supportive presence, intrusiveness, and questioning style). Detailed results of the bootstrapping analyses with parenting strategies as a mediator in the relation between gender and social competence are provided in Supplementary Table 1.

#### Social Behavioral Competence at School

There was no mediation effect for gender on social behavioral competence at school via any of the parenting strategies. Standardized indirect effects via SP (*b* = −0.34, *SE* = 0.48, 95% CI [−1.88, 0.27]) and intrusiveness (*b* = −0.04, *SE* = 0.56, 95% CI [−1.28, 1.08]) were non-significant. Nor were standardized indirect effects via parental questioning style (*b*_total_ = 0.07, *SE* = 0.30, 95% CI [−0.28, 1.12]; *b*_ratio_ = < 0.01, *SE* = 0.24, 95% CI [−0.53, 0.55]).

#### Social Behavioral Competence at Home

There was no mediation effect for gender on social behavioral competence at home via any of the parenting strategies. Standardized indirect effects via SP (*b* = < −0.01, *SE* = 0.23, 95% CI [−0.60, 0.42]) and intrusiveness (*b* = < −0.01, *SE* = 0.27, 95% CI [−0.61, 0.55]) were non-significant. Nor were standardized indirect effects via parental questioning style (*b_total_* = −0.05, *SE* = 0.33, 95% CI [−1.07, 0.40]; *b_ratio_* = 0.01, *SE* = 0.35, 95% CI [−0.67, 0.81]).

#### Social Cognition

There was no mediation effect for gender on social cognition via any of the parenting strategies. Standardized indirect effects via SP (*b* = −0.25, *SE* = 0.54, 95% CI [−1.84, 0.52]) and intrusiveness (*b* = −0.05, *SE* = 0.50, 95% CI [−1.13, 0.95]) were non-significant. Nor were standardized indirect effects via parental questioning style (*b_total_* = −0.08, *SE* = 0.36, 95% CI [−1.15, 0.44]; *b_ratio_* = −0.04, *SE* = 0.26, 95% CI [−0.82, 0.30]).

## Moderation Analyses: Differential Susceptibility or Diathesis-Stress

Results of the most parsimonious model of each hierarchical regression analysis of SP, intrusiveness and questioning style explaining each aspect of social competence are presented in Tables 3, 4. In each regression analysis the following models were tested: (model 1) the aspect of parenting strategy, verbal ability, gender and age were included; (model 2) the quadratic term of parenting strategy was added; (model 3) the interaction term between parenting strategy and gender was added; (model 4) the interaction between parenting strategy and age was added; (model 5) the interaction between gender and age and the three-way interaction between parenting strategy, gender and age were added. The predicted *R^2^* value of each model was reasonably close to the corresponding adjusted *R^2^* values, indicating that overfitting was not an issue.

**Table 3:** Hierarchical regression analysis results of most parsimonious models for parental supportive presence and intrusiveness with aspects of child social competence.

		Social behavior						Social cognition		
		School (*n* = 89)			Home (*n* = 91)			SCST total (*n* = 98)		
Independent variables		b (SE)	95% CI		b (SE)	95% CI		b (SE)	95% CI	
Supportive presence			Low	Up		Low	Up		Low	Up
Intercept		41.53 (1.32)	38.83	44.16	53.03 (1.32)	50.42	55.69	27.14 (1.47)	24.26	29.94
M1	SP	1.92 (1.08)^†^	−0.04	3.93	0.06 (1.13)	−1.95	2.17	2.51 (1.20)^∗∗∗^	0.38	4.59
	Age	−1.63 (1.17)	−4.17	.72	−1.44 (1.25)	−3.61	1.11	4.63 (1.32)^∗∗∗^	1.96	7.29
	Gender	7.61 (2.00)^∗∗∗^	3.76	11.26	4.82 (2.04)^∗∗∗^	0.81	8.50	3.72 (2.22)^†^	−0.74	8.32
	Verbal ability	0.39 (0.13)^∗∗∗^	0.15	.64	0.24 (0.13)^†^	−0.05	.50	0.48 (0.14)^∗∗∗^	0.18	0.77
*Adj. R^2^ / Pred. R^2^*		0.21/0.17			0.06/0.02			0.46/0.42		
Δ *R*^2^ / *F* Δ *R*^2^		25/6.87^∗∗∗^			0.10/2.32^†∗∗^			0.48/21.22^∗∗∗^		
**Intrusiveness**										
Intercept		41.76 (1.30)	39.12	44.40	53.01 (1.31)	50.13	55.81	28.33 (1.83)	24.62	32.03
M1	I	−2.64 (1.08)^∗∗∗^	−4.95	−0.50	−0.80 (1.16)	−3.48	1.69	−2.84 (1.63)^†^	−6.05	0.06
	Age	−1.24 (1.17)	−3.67	1.09	−1.34 (1.25)	−3.61	1.32	5.76 (1.38)^∗∗∗^	2.56	8.53
	Gender	7.32 (1.96)^∗∗∗^	3.49	10.97	4.82 (2.03)^∗∗∗^	0.72	8.62	3.43 (2.19)	−1.00	7.82
	Verbal ability	0.36 (0.12)^∗∗∗^	0.12	.60	0.24 (0.13)^†^	−0.03	1.69	0.36 (0.15)^∗∗∗^	0.06	0.71
M2	I^2^							−0.74 (1.38)	−3.17	1.83
M3	I × Gender							1.92 (2.49)	−2.61	6.45
M4	I × Age							−2.15 (1.03)^∗∗∗^	−3.87	−0.52
*Adj. R^2^/Pred. R^2^*		0.24/0.19			0.06/0.02			0.47/0.43		
Δ *R*^2^ /*F* Δ *R*^2^		27/7.76^∗∗∗^			10/2.45^†^			0.02/4.39^∗∗∗^		

*M1: first model with linear independent variable, age, gender, and receptive verbal ability; M2: second model quadratic independent variable added; M3: third model adding linear interaction with gender; M4: fourth model adding linear interaction with age; M5: fifth model adding linear interaction age by gender and three-way interaction with age by gender. Variables marked with superscript 2s are curvilinear variables. Adjusted R^2^ and predicted R^2^ of the most parsimonious model is reported. Δ R^2^: change in R^2^ in comparison with the previous model. F Δ R^2^: F for change in R^2^ in comparison with the previous model. B: unstandardized coefficient. SE: standard error. ^†^p < 0.10; ^∗^p < 0.05; ^∗∗^p < 0.01; ^∗∗∗^p < 0.001.*

**Table 4:** Hierarchical regression analysis results of most parsimonious models for parental questioning style with aspects of child social competence.

		Social behavior						Social cognition		
		School (*n* = 89)			Home (*n* = 91)			SCST total (*n* = 98)		
Independent variables		B (SE)	95% CI		b (SE)	95% CI		b (SE)	95% CI	
Total questions			Low	Up		Low	Up		Low	Up
Intercept		41.60 (1.34)	38.81	44.46	54.72 (1.51)	51.64	58.13	28.14 (1.68)	24.63	31.41
M1	Questions	−1.16 (1.35)	−3.58	1.66	0.20 (2.03)	−3.93	7.91	4.93 (2.07)^∗∗∗^	0.97	9.15
	Age	−1.86 (1.20)	−4.62	0.76	−1.89 (1.47)	−4.39	0.89	5.11 (1.36)^∗∗∗^	1.90	8.38
	Gender	7.21 (2.02)^∗∗∗^	3.21	11.06	3.24 (2.15)	−1.29	7.52	3.56 (2.07)	−0.88	7.86
	Verbal ability	0.35 (0.13)^∗∗∗^	0.11	0.60	0.27 (0.13)^∗∗∗^	−0.04	0.53	0.49 (0.15)^∗∗∗^	0.20	0.79
M2	Q^2^				−1.29 (1.33)	−4.37	0.40	−1.19 (1.11)	−3.38	1.27
M3	Q × Gender				2.75 (2.72)	−3.93	7.91	−5.55 (2.68)^∗∗∗^	−10.70	−1.33
M4	Q × Age				1.55 (1.81)	−1.61	4.33			
M5	Age × Gender				1.55 (1.86)	−2.13	4.51			
	Q × Age × Gender				−4.72 (2.12)^∗∗∗^	−8.92	−0.93			
*Adj. R^2^ / Pred. R^2^*		0.19/0.14			0.13/0.07			45/0.41		
Δ *R*^2^ /*F* Δ *R*^2^		0.23/6.09^∗∗∗^			0.06/3.21^∗∗∗^			0.02/4.28^∗∗∗^		
**Ratio open-closed**										
Intercept		41.64 (1.35)	38.84	44.46	53.00 (1.30)	50.27	55.61	27.22 (1.50)	24.27	30.04
M1	Ratio	−0.14 (1.12)	−2.48	2.15	1.43 (1.10)	−0.53	3.31	0.42 (1.24)	−2.05	2.71
	Age	−1.71 (1.20)	−4.43	0.78	−1.43 (1.23)	−3.63	1.07	4.55 (1.35)^∗∗∗^	1.65	7.61
	Gender	7.28 (2.03)^∗∗∗^	3.42	10.98	4.80 (2.02)^∗∗∗^	0.60	8.64	3.51 (2.27)	−1.05	8.32
	Verbal ability	0.38 (0.13)^∗∗∗^	0.13	0.64	0.24 (0.13)^†^	−0.03	0.48	0.45 (0.15)^∗∗∗^	0.13	0.76
*Adj. R^2^/Pred. R^2^*		0.18/0.13			0.07/0.04			0.43/0.39		
Δ *R*^2^/*F* Δ *R*^2^		0.22/05.86^∗∗∗^			0.12/2.79^∗∗∗^			0.45/19.27^∗∗∗^		

*M1: first model with linear independent variable, age, gender and receptive verbal ability; M2: second model quadratic independent variable added; M3: third model adding linear interaction with gender; M4: fourth model adding linear interaction with age; M5: fifth model adding linear interaction age by gender and three-way interaction with age by gender. Variables marked with superscript 2s are curvilinear variables. Adjusted R^2^ and predicted R^2^ of the most parsimonious model is reported. Δ R^2^: change in R^2^ in comparison with the previous model. F Δ R^2^: F for change in R^2^ in comparison with the previous model. B: unstandardized coefficient. SE: standard error. ^†^p < 0.10; ^∗^p < 0.05; ^∗∗^p < 0.01; ^∗∗∗^p < 0.001.*

### Parental Sensitivity

#### Social Behavioral Competence at School

Models 2 to 5 were no significant improvement over Model 1 (all *p*_*F* Δ *R*2_ > 0.05), suggesting that neither gender nor age significantly moderated the association between SP or intrusiveness and social behavioral competence at school. A main effect of intrusiveness was found. Higher intrusiveness was significantly related to fewer social behavioral skills at school in the whole sample (β = −0.24, *p* = 0.01). The threshold for statistical significance was not achieved for the association between SP and social behavioral skills at school (β = 0.17, *p* = 0.07). However, this trend suggests that parents who are more supportive tend to have children who have slightly better social behavioral skills at school.

#### Social Behavioral Competence at Home

Models 2 to 5 were no significant improvement over Model 1 (all *p*_*F* Δ *R*2_ > 0.05), suggesting that neither gender nor age significantly moderated the association between SP or intrusiveness and social behavioral competence at home. Nor were there any significant associations between SP or intrusiveness and social behavioral competence at home.

#### Social Cognition

**FIGURE 2:**
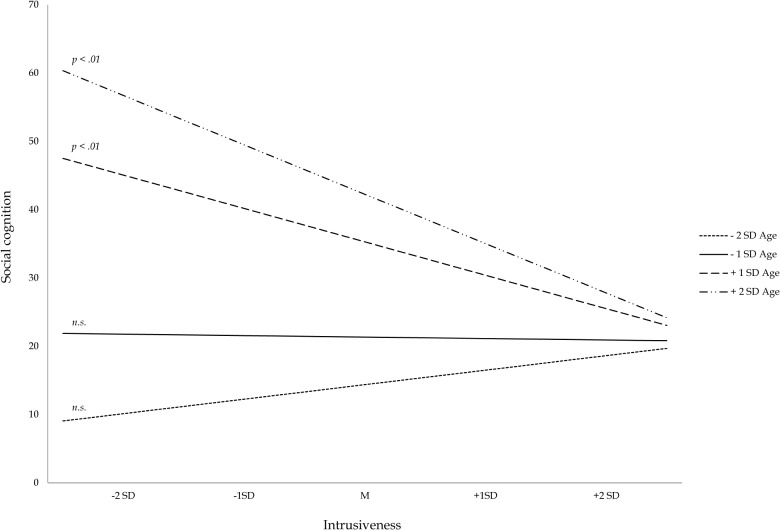
Moderation effect of age on the relation between parental intrusiveness and social cognitive competence.

A significant age interaction effect was found for the association between intrusiveness and social cognition. The relation between intrusiveness and social cognition was best described by including age as a moderator (Model 4 *p*_*F* Δ *R*2_ = 0.03, *BF*_10_ = 3.08; see also Figure 2). Bayesian analyses indicated substantial evidence for an age interaction effect. *Post hoc* probing showed that a lower level of intrusiveness was significantly associated with better social cognitive skills in older children (+2 *SD* β = 0.46, *p* < 0.01; +1 *SD* β = 0.29, *p* < 0.01), but not in younger children (*p* > 0.05). Gender did not significantly moderate the relation between SP or intrusiveness and social cognition. A main effect of SP was found. Higher SP was related to better social cognition in the whole age-range and this relation was similar for boys and girls (β = 0.16, *p* = 0.03).

### Parental Questioning Style

#### Social Behavioral Competence at School

Models 2 to 5 were no significant improvement over Model 1 (all *p*_*F* Δ *R*2_ > 0.05), suggesting that neither gender nor age significantly moderated the association between parental questioning style and social behavioral competence at school. Nor were there any significant main effects between parental questioning style and social behavioral competence at school.

#### Social Behavioral Competence at Home

**FIGURE 3:**
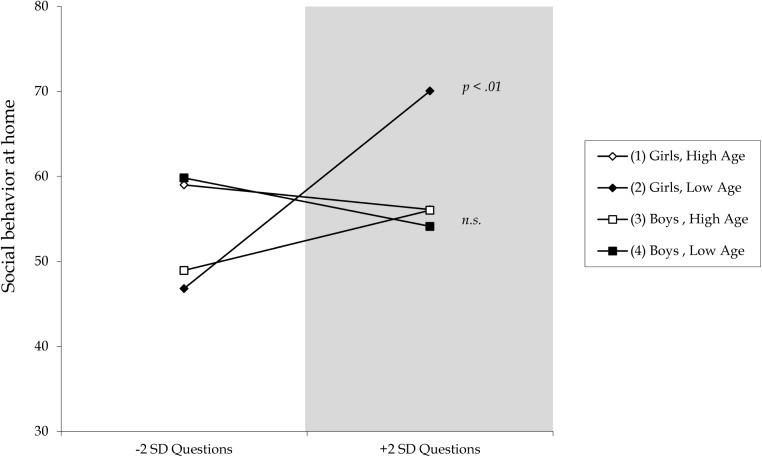
Three-way gender by age interaction effect on the relation between total questions asked and social behavioral competence at home. Gray shaded area denotes region where the lines significantly differ.

A significant three-way interaction was found for total questions when considering gender and age (Model 5 *p*_*F* Δ *R*2_ = 0.04, *BF*_10_ = 2.43). Bayesian analyses indicated anecdotal evidence for a gender by age interaction effect, but no substantial evidence for the absence of such an effect. *Post hoc* probing (see Figure 3) showed that only in younger girls, was having a parent who asks more questions related to better social behavioral skills at home (β = 0.60, *p* < 0.01). The lower-bound RoS was below −2 *SD* on questions (RoS = 3.06–0.52; see shaded region only at the high end of total questions in Figure 3), suggesting only the “for-better” side of the differential susceptibility model is supported; the exact opposite of the diathesis-stress model. No significant associations between social behavioral skills at home and open- versus closed- ended questions ratio score were found.

#### Social Cognition

**FIGURE 4:**
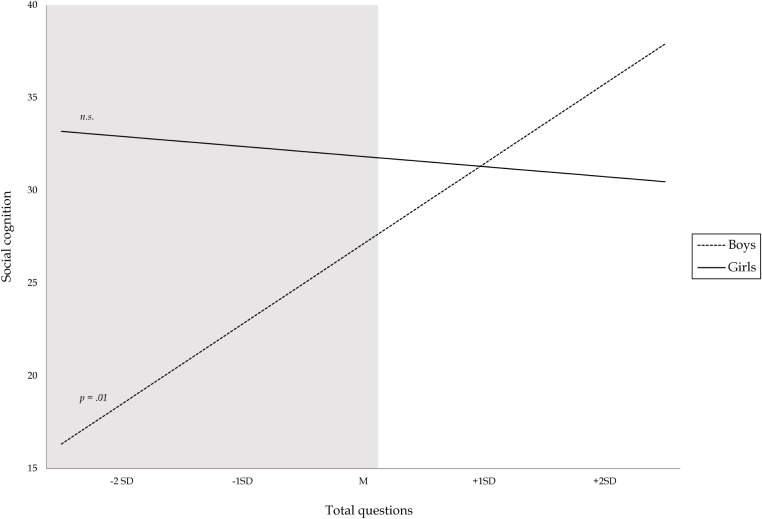
Moderation effect of gender on the relation between total questions asked and social cognitive competence. Gray shaded area denotes region where the two lines significantly differ.

A significant gender interaction effect was found for total questions. The relation between the total amount of questions asked by parents and social cognition was best described by including gender as a moderator (Model 3 *p*_*F* Δ *R*2_ = 0.04, *BF*_10_ = 6.18). Bayesian analyses indicated substantial evidence for a gender interaction effect. *Post hoc* probing (see Figure 2) showed that asking fewer questions was only significantly related to poorer social cognition in boys (β = 0.29, *p* = 0.01). The upper-bound RoS was above +2 *SD* on questions (RoS = −0.17–16.93; see shaded region only at the low end of total questions in Figure 4), suggesting these results are consistent with the diathesis stress model. No significant associations between social cognition and open- versus closed-ended questions ratio score were found.

## Discussion

The aim of the current study was to investigate whether aspects of parenting strategies, i.e., supportive presence, intrusiveness and questioning style, are associated with child social competence during the early school years and to what extent (1) these parental strategies mediate the relation between gender and social competence, in line with a differential socialization model; and (2) whether gender and age moderate the relation between parenting strategies and social competence, distinguishing between the differential susceptibility and diathesis stress models. This study showed that parenting strategies did not mediate the relation between gender and social competence, suggesting gender differences in social behavioral competence could not be explained by differential socialization of boys and girls. Social behavioral competence at school was related to intrusiveness and social cognition was related to supportive presence, while controlling for verbal ability. Gender moderated the association between the amount of questions asked by parents and children’s social cognition. Only boys of parents who asked fewer questions showed lower levels of social cognition, in line with the diathesis-stress model. Furthermore, only in older children, lower levels of intrusiveness were related to better social cognition, and only in younger girls, having a parent who asks more questions was related to better social skills at home.

### Differential Socialization

The gender differences in social behavioral competence could not be explained by parental differential socialization as far as parental sensitivity or questioning style are concerned. Parents did not interact with their sons and daughters in a different way. It was expected that gender-differentiated parenting would only be true for some, but not all parenting strategies that have been associated with social competence. In particular, we hypothesized that parents would ask more questions to their daughters than to their sons, in line with the meta-analysis by Leaper et al. (1998), which we could not confirm. Even though differential socialization of sons and daughters has not been consistently found in meta-analyses, all concluded that gender differences in parenting decrease with age (Lytton and Romney, 1991; Leaper et al., 1998; Endendijk et al., 2016). For instance, differential verbal socialization was especially apparent in mothers of toddlers, compared to mothers of school-aged children (Leaper et al., 1998). Similarly, differential socialization in emotion talk was only found in 4-year-olds and not 6-year-olds (Aznar and Tenenbaum, 2015), suggesting differential socialization by parents may change with age. In this study, a somewhat older age-range of 4- to 8-year-old children was studied. Even though differential socialization may influence social competence during early childhood, based on this study we might conclude that after age four, gender differentiated parenting appears to diminish with respect to sensitivity and questioning style.

### Diathesis-Stress or Differential Susceptibility

Higher levels of parental support were significantly related to better social cognitive skills in their children and tended to relate to better social behavioral skills at school. Higher levels of parental intrusiveness were significantly related to worse social behavioral skills at school in their children. These findings are in line with previous studies, suggesting that parental sensitivity is linked to the development of social competence (e.g., Lengua et al., 2007; Spinrad et al., 2007; Barnett et al., 2012). It was hypothesized that the relative influence of parenting on social competence would decrease with age, in line with the diminished need for structure as children start school, which we could not confirm. Surprisingly, lower levels of intrusiveness were only related to better social cognition in older children, suggesting that how intrusiveness matters in relation to social cognition varies with age. This is consistent with the findings of Landry et al. (2000) in a slightly younger sample, who showed that parents providing verbal structure had a positive effect on cognitive and social development, but that this effect reversed after age four. Perhaps children still require some structure from their parents with regard to more sophisticated developmental tasks, such as self- and third party perspective taking, even after they have started school. As such, higher levels of intrusiveness may be an appropriate parenting strategy with regard to social cognition in younger children, while lower levels of intrusiveness become more adaptive as children age. In contrast with our hypothesis, these associations were not stronger for boys. Studies reporting stronger associations between parental sensitivity and social competence in boys generally focus on the early childhood years (for a review, see Bornstein, 2005). As the relation between parenting and child behavior changes with age (Bradley et al., 2015) and individual differences in environmental susceptibility may vary with age (Ellis et al., 2011), this finding may be due to the somewhat older 4- to 8-year-old age range in the current study. Rather, our data suggest that during the early school years, parental sensitivity may be related to child social competence irrespective of gender.

In line with a diathesis-stress model, worse social cognitive skills in boys were related to parents asking fewer questions. This is consistent with research on the development of child behavioral problems, suggesting boys are more vulnerable to adverse parenting effects than girls (e.g., Calkins, 2002; Crick and Zahn-Waxler, 2003; Barnett and Scaramella, 2013). Surprisingly, the gender by age interaction effect found in this study was only partially consistent with the differential susceptibility model and the exact opposite of the diathesis stress model. Only in younger girls was having a parent who asks more questions related to better social skills at home. In other words, only the “for-better” side of the differential susceptibility model was supported. Rather than parental questioning style protecting young girls from showing worse social behavioral skills, this suggests that girls functioned better than younger boys when parents asked more questions. This opposite of vulnerability has been described as vantage sensitivity (Manuck, 2011), suggesting girls may have an advantage to thrive under optimal parenting conditions compared to boys. However, Bayesian analyses did not indicate clear evidence for a gender by age interaction effect nor for the absence of such an effect, which suggests that longitudinal studies are better equipped to disentangle these associations. Parents asking more questions to their children may represent an overall better parental verbal ability, an increased awareness of the importance of having rich verbal communication with their child, or more encouragement of their children’s learning. Even though parental questioning style was not significantly associated with parents’ educational level in this study, parents were less likely to have a low educational attainment. Based on our study we cannot conclude with any certainty the rationale behind parents’ questioning style and believe this to be a potential avenue for further research.

Even though the gender by age interaction effect seems counterintuitive, as relations were expected to be stronger for boys (Leaper, 2002), they may be explained by a transactional model of parent–child interaction, indicating a reciprocal relation between child behavior and parenting strategies (Sameroff, 2009). Parental questioning style may stimulate the development of social competence, but more socially competent children, and in particular girls, may also evoke more questions from parents because they are more responsive and cooperative (Barnett et al., 2012). Due to the cross-sectional nature of the current study, no definite answer on causality in these associations can be given. Perhaps parents who perceive their daughters as more social ask them more questions than they ask their sons, as overall parents rated their daughters as more social than their sons. This would be substantiated by the finding in this study that teachers also rated girls as being more socially competent, but that there were no gender differences in the association between parental questioning style and social behavior at school. Although speculative, this would suggest the nature of this relation relies more on how parents perceive their daughters than on how their questioning style influences their child’s social behavior.

Nonetheless, these findings support the idea that only some aspects of parenting strategies have a differential effect on the development of social competence of boys and girls. More specifically, only parents’ questioning style and not aspects of parental sensitivity seems to have gender-differentiated associations with social competence in young school-aged children. Furthermore, these findings underscore the importance of formally testing moderation effects when distinguishing the differential susceptibility model from the diathesis-stress model (Roisman et al., 2012). Drawing conclusions based on visual inspection of the interaction plots may have led to the false assumption that data were consistent with the differential susceptibility model.

### Strengths and Limitations

Several limitations of the current study need to be acknowledged. Parents may have acted differently than their usual self during the joint-activity tasks as a consequence of being videotaped. However, due to the relatively natural observation conditions in the home, it is unlikely that the nature of the interactions was considerably misrepresented (Gardner, 2000). Secondly, in the current study it was impossible to differentiate between fathers’ and mothers’ interactive style due to low occurrence of participating fathers (10%), possibly obscuring parental gender effects on differential socialization. Thirdly, children from only two Dutch schools in the same provincial region participated in this study, which may limit the generalizability of our findings. In addition, the current sample did not accurately represent families from a lower educational background, as the number of parents with a low educational level was underrepresented (7.5% compared to expected 33.6% in Dutch 25–45-year-olds; Central Bureau for Statistics [CBS], 2013). Fourthly, several relatively complex analyses were conducted using a modest sample size. However, cross-validation by examining confidence intervals based on 5000 bias-corrected bootstraps, comparing predicted *R^2^* values with adjusted *R^2^* values to avoid overfitted models, and Bayesian analyses raised no major concerns. Finally, the current study assessed associations between parental strategies and child social competence cross-sectionally, and no inferences concerning developmental changes within children or causality can be made.

Strengths of this study include the use of multiple well-validated social competence measures and the use of different informants. Moderation effects were thoroughly investigated and controlled for non-linearity of effects as recommended by Roisman et al. (2012). Verbal ability was also controlled for in this study, indicating our findings are not due to gender differences in language skills. Furthermore, observed parenting behaviors were coded objectively and included aspects of parental sensitivity as well as parent–child verbal interaction.

In sum, our results indicate that parent–child questioning style and not aspects of parental sensitivity seems to have gender-differentiated associations with social competence in school-aged children, while parents do not treat their sons and daughters differently at this age. In boys, asking fewer questions was associated with worse social cognitive skills, in line with a diathesis-stress model. These findings suggest opportunities to educate parents to be more supportive in general, become less intrusive as their children mature and to ask more questions, especially to their sons, which may enhance social competence. Furthermore, our findings underscore the importance of formally testing moderation effects as well as testing for non-linearity to avoid misleading interactions (Ganzach, 1997; Holmbeck, 2002; Roisman et al., 2012). For instance, drawing conclusions based on visual inspection of the interaction plots instead of probing interaction effects may lead to false assumptions which may seriously hinder the interpretation of study results. Future studies assessing moderation effects should also consider curvilinear effects and *post hoc* probing before interpreting their results.

## Author Contributions

AS carried out the data collection, conducted the analyses, and wrote the manuscript with support from the other authors. MD helped to supervise the project. HS devised the project. All authors discussed the results and commented on the manuscript and provided critical feedback and helped to shape the research, analysis and manuscript.

## Conflict of Interest Statement

The authors declare that the research was conducted in the absence of any commercial or financial relationships that could be construed as a potential conflict of interest.

## Footnotes

1 http://www.qualtrics.com/
