# Supplementary material for: Linking Parenting and Social Competence in School-Aged Boys and Girls: Differential Socialization, Diathesis-Stress, or Differential Susceptibility?
Source: Front Psychol. 2019 Jan 15;9:2789. doi: 10.3389/fpsyg.2018.02789 (PMC6340968; doi:10.3389/fpsyg.2018.02789)
Supplement: Supplementary file 1 [file Table_1.DOCX]

Supplementary Material

Linking parenting and social competence in school-aged boys and girls: Differential socialization, diathesis-stress or differential susceptibility?

A.M. Spruijt*, M.C. Dekker, T.B. Ziermans, H. Swaab

*** Correspondence:** Andrea Spruijt: [a.m.spruijt.2@fsw.leidenuniv.nl](mailto:a.m.spruijt.2@fsw.leidenuniv.nl)

|  | | **Social behavior at school**  **(n=89)** | | |  | **Social behavior at home**  **(n=91)** | | |  | **Social cognition  (N=98)** | | |
| --- | --- | --- | --- | --- | --- | --- | --- | --- | --- | --- | --- | --- |
|  | |  | 95% CI | |  |  | 95% CI | |  |  | 95% CI | |
| Mediator | | *b (SE)* | Lower | Upper |  | *b (SE)* | Lower | Upper |  | *b (SE)* | Lower | Upper |
| Total effect | | 7.28 (2.02)*** | 3.26 | 11.29 |  | 4.81 (2.02)* | .79 | 8.84 |  | 3.47 (2.26) | -1.01 | 7.95 |
| Age | | -1.71 (1.19) | -4.07 | .65 |  | -1.44 (1.24) | -3.90 | 1.02 |  | 4.57 (1.34)** | 1.90 | 7.24 |
| Verbal ability | | .38 (.13)** | .13 | .63 |  | .24 (.13) | -.02 | .50 |  | .45 (.14)** | .16 | .74 |
|  | |  |  |  |  |  |  |  |  |  |  |  |
| **Supportive presence (SP)** | |  |  |  |  |  |  |  |  |  |  |  |
|  | Direct effect Gender - SP | -.17 (.20) | -.57 | .23 |  | -.07 (.19) | -.46 | .32 |  | -.10 (.19) | -.48 | .28 |
|  | Direct effect Gender - SC | 7.61 (2.00)*** | 3.63 | 11.59 |  | 4.82 (2.04)* | .77 | 8.89 |  | 3.72 (2.22) | -.69 | 8.13 |
|  | Indirect effect (mediation) | -.34 (.48) | -1.88 | .27 |  | < -.01 (.23) | -.60 | .42 |  | -.25 (.54) | -1.84 | .52 |
| **Intrusiveness (I)** | |  |  |  |  |  |  |  |  |  |  |  |
|  | Direct effect gender - I | .01 (.20) | -.38 | .41 |  | <.01 (.19) | -.37 | .38 |  | .02 (.18) | -.34 | .38 |
|  | Direct effect gender - SC | 7.32 (1.96)*** | 3.41 | 11.22 |  | 4.82 (2.03)* | .78 | 8.85 |  | 3.52 (2.22) | -.89 | 7.92 |
|  | Indirect effect (mediation) | -.04 (.56) | -1.28 | 1.08 |  | < -.01 (.27) | -.61 | .55 |  | -.05 (.50) | -1.13 | .95 |
| **Total questions (TQ)** | |  |  |  |  |  |  |  |  |  |  |  |
|  | Direct effect gender - TQ | -.06 (.16) | -.39 | .26 |  | -.05 ( .17) | -.37 | .28 |  | -.05 (.16) | -.37 | .27 |
|  | Direct effect gender - SC | 7.21 (2.02)*** | 3.26 | 11.29 |  | 4.87 (2.03)* | .83 | 8.90 |  | 3.56 (2.25) | -.91 | 8.03 |
|  | Indirect effect (mediation) | .07 (.30) | -.28 | 1.12 |  | -.05 (.33) | -1.07 | .40 |  | -.08 (.36) | -1.15 | .44 |
| **Ratio questions (RQ)** | |  |  |  |  |  |  |  |  |  |  |  |
|  | Direct effect gender - RQ | <.01 (.20) | -.39 | .39 |  | <.01 (.20) | -.38 | .40 |  | -.08 (.19) | -.46 | .29 |
|  | Direct effect gender | 7.28 (2.03)*** | 3.24 | 11.32 |  | 4.80 (2.02)* | .80 | 8.81 |  | 3.51 (2.27) | -1.00 | 7.95 |
|  | Indirect effect (mediation) | <.01 (.24) | -.53 | .55 |  | .01 (.35) | -.67 | .81 |  | -.04 (.26) | -.82 | .30 |

Table 1.
Bootstrapping analyses results with parenting strategies as a mediator in the relation between gender and social competence.

*Note.* Results based on 5000 bootstrapped samples. SC = Social competence (dependent variable). 95% CI = bias-corrected and accelerated confidence intervals, with **p* <.05 when range lower‑upper CI does not include zero.
